# Esophageal Perforation into the Pericardium in a 3-Year-Old Child with Esophageal Stricture: A Rare Complication Following Esophageal Dilatation

**DOI:** 10.1055/s-0042-1756207

**Published:** 2022-09-02

**Authors:** Abdulrahman Nasser, Raif Nassir, Muhammad Younas Awan, Mohammad Anas AlShawa, Zakaria Habib

**Affiliations:** 1 College of Medicine, Taibah University, Al Madinah Al Monawrrah, Saudi Arabia; 2 Department of Pediatric Surgery, King Salman Medical City, Al Madinah Al Monawrrah, Saudi Arabia; 3 Department of Surgery, King Faisal Specialist Hospital and Research Centre, Riyadh, Saudi Arabia

**Keywords:** esophageal dilatation, pneumomediastinum, pericardial perforation

## Abstract

Perforation of the esophagus during dilatation is a rare complication that might cause mortality. We present the report of a 3-year-old girl who was diagnosed with B cell acute lymphoblastic leukemia at 17 months of age. She experienced a complicated clinical course after chemotherapy was initiated, which included mucositis and acute pericarditis. She later developed an acquired esophageal stricture and tracheoesophageal fistula, which were managed with resection and primary anastomosis when she was in remission. Postoperatively, the patient developed a leak, which was treated conservatively. She subsequently developed a stricture that was treated successfully. On the fourth dilatation attempt and after she was sent home, she presented with persistent vomiting and low-grade fever and became vitally unstable on the same day, after stabilization, upper gastroenterology contrast revealed contrast filling the pericardium. She was managed conservatively with close observation and serial echocardiograms and then discharged home on day 18 in good condition after complete resolution of the pericardial effusion.

## Introduction

Perforation of the esophagus during dilatation is a rare complication that might lead to mortality in the pediatric patients, 1 It is crucial for the patient's outcome that the perforation is recognized, treated with quick intervention, and close observation. 2 To our knowledge, esophageal perforation into the pericardium during dilation has not been reported in the literature; we think that perforation into the pericardium might carry a higher risk of morbidity and mortality, Therefor, we share our experience in diagnosing and managing the patient.

## Case Description

We present a case of a 3-year-old girl who was diagnosed with B cell acute lymphoblastic leukemia at the age of 17 months, who underwent a complicated course after chemotherapy was initiated, including febrile neutropenia, viral gastroenteritis, severe mucositis, esophagitis, and septic shock due to acute pericarditis. Soon after, she started to experience worsening dysphagia, regurgitation, and cough. Esophagogram revealed multiple segments of esophageal narrowing at the mid-esophagus with a diameter of 7-mm and a 3-cm long segment in the distal esophagus with a diameter of 3 mm, confirming an acquired esophageal stricture.

Four trials of dilatation were attempted. The first was undertaken through endoscopy but failed to bypass the stricture or dilate it, a gastrostomy tube for feeding was inserted surgically at the same setting. A second dilatation was performed by an interventional radiologist using fluoroscopy guidance of an 8-mm balloon. Although it was successful, it revealed a fistula communication between the esophagus and the right main bronchus. We considered applying a glue to treat bronchoesophageal fistula but the idea was dismissed as the third trial was aborted because the interventional radiologist was unable to bypass the stricture. The fourth trial was attempted by a pediatric surgeon using a rigid esophagoscope but failed to dilate the stricture, which had progressed to almost complete obliteration of the esophageal lumen. The multidisciplinary team discussed with the patient's family and decided to postpone the dilatations, depend on gastrostomy feeding, and watch for any signs of chest infections because the risks outweighed the benefits of surgical intervention at this point. The patient did not appear to have any complications from swallowing her saliva nor did she had complications from the fistula, possibly due to the structure obliterating it. After the patient was in remission, surgical correction of the esophageal stricture was achieved with primary anastomosis at the age of 2 years and 7 months. The surgical correction was attempted through a right thoracotomy incision and a retropleural approach into the mediastinum, the proximal part of the esophagus was identified and found to be dilated, the fistula was ligated and transected, and resection of the stenotic part of the esophagus with the fistula was achieved, The distal part was mobilized until reaching the esophageal hiatus. Anastomosis was achieved without tension, transanastomotic tube was inserted, and no flap was placed around the anastomosis, The procedure duration was 150 minutes.

Postoperatively, the patient developed a leak evident clinically by tachypnea and saliva secretion drained through the intrapleural chest drain and radiologic signs of pleural effusion. She was treated conservatively with complete fasting, parenteral nutrition, and observation, and required antibiotics coverage with ceftazidime and clindamycin for 7 days. She was then upgraded to vancomycin and piperacillin/tazobactam to complete 21 days. The patient responded to conservative management, and repeated esophagogram revealed no signs of leak or fistula; however, a 4-mm stricture correlated to the area of anastomosis was found.

Eventually, she required nine interval dilatations, which were achieved using rigid esophagoscopy and Savary-Gilliard serial dilatations over a guidewire. We started with 5-Fr on her initial dilatation and gradually upgraded through the series of dilatations. On the fourth esophageal dilatation, a size 15-Fr was used with minimal resistance and without intraoperative complications. The patient tolerated the procedure and was discharged home on the same day.

**Fig. 1:**
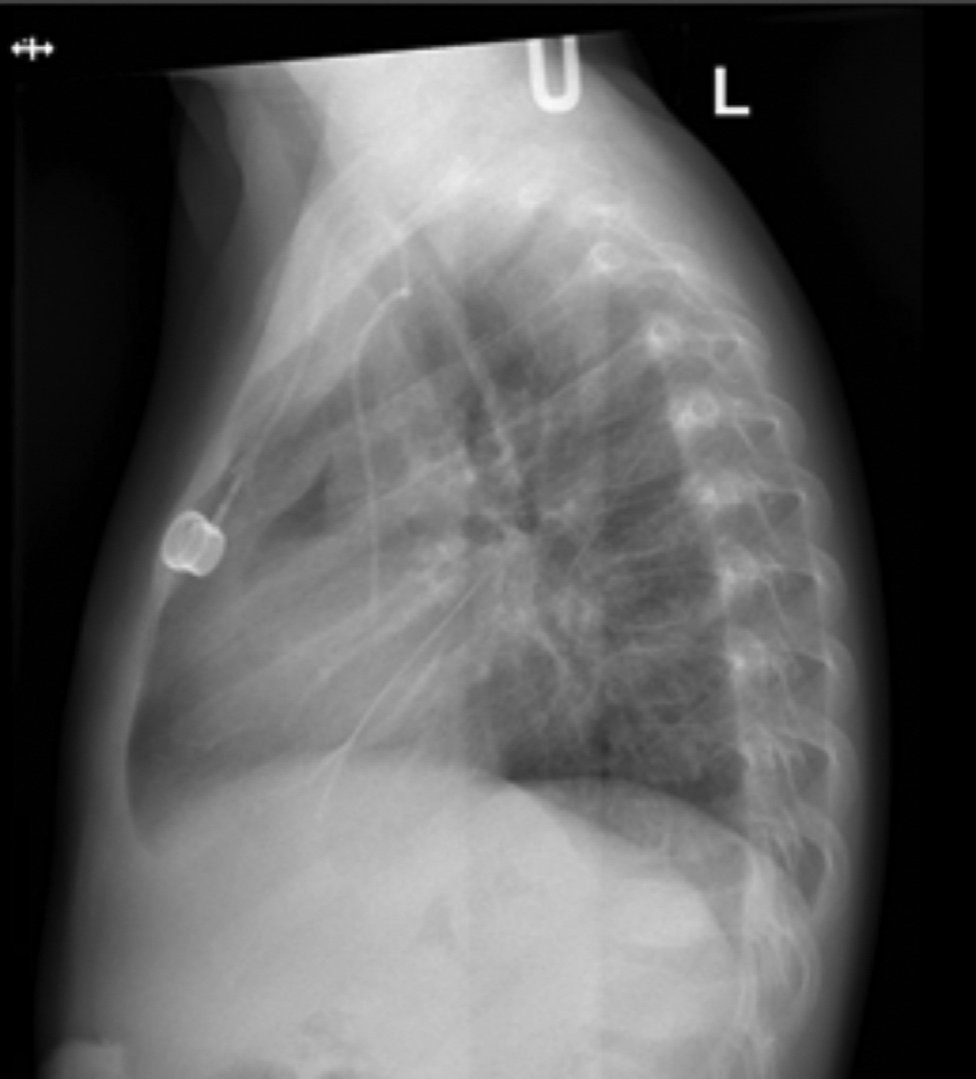
Lateral view of chest X-ray on day 0 postesophageal dilatation demonstrating lucency in the middle mediastinum, which is suggestive of esophageal perforation.

**Fig. 2:**
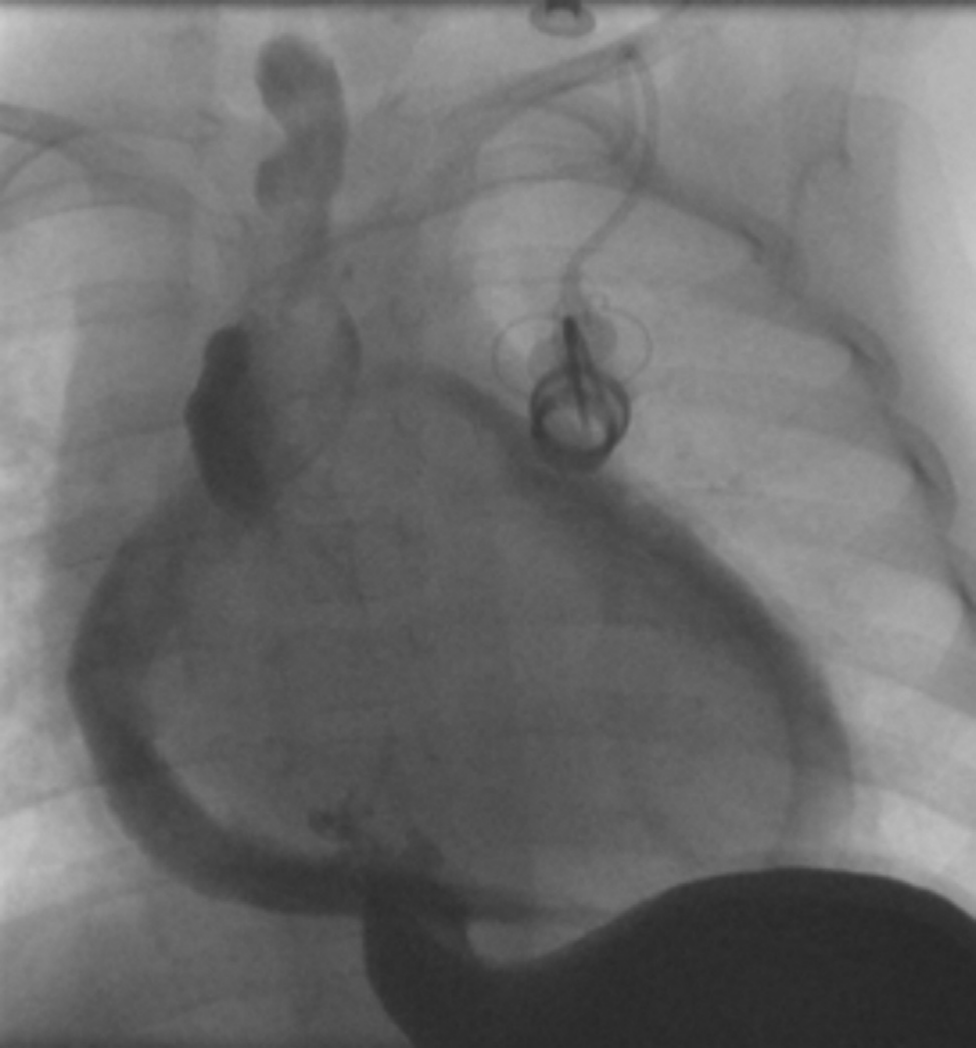
On day 1 postesophageal dilatation, the lining of the pericardium is visualized by the extravasation of the contrast during an upper gastrointestinal study.

On day 0, she presented to our emergency department with persistent vomiting and lethargy and was found to have high blood pressure. She then developed tachycardia, tachypnea, and low-grade fever. Her initial white blood cell count was 20.50, which increased to 26.33. Septic workup was obtained, including a chest X-ray ( Fig. 1 ) that showed signs of pneumomediastinum in both anteroposterior and lateral views and which were more evident in the lateral chest view. The patient became hemodynamically stable after the start of initial treatment, which included intravenous hydration and broad-spectrum antibiotics. A water-soluble contrast study revealed contrast lining the pericardium, suggesting a perforation and resulting in pericardial effusion ( Fig. 2 ). Urgent echocardiogram showed small circumferential pericardial effusion with no evidence of cardiac tamponade. Septic cultures were negative. We elected to continue conservative management and close observation, including total parenteral nutrition, but did not insert a nasogastric tube, choosing instead to use the existing gastrostomy tube for stomach drainage. On day 2, the patient became tachycardic and tachypneic and required oxygen support by nasal cannula. A repeat echocardiogram showed moderate-sized circumferential pericardial effusion, which had increased in size as compared with the previous study but did not show signs of external compression of the right ventricle or right atrium. However, inferior vena cava and hepatic veins were dilated with absent respiratory collapsibility and reversal of flow was present in the hepatic veins. Doppler was suggestive of high right atrium pressure. On day 4, the patient improved clinically with normal vitals except for mild tachypnea on minimal oxygen support. White blood cell counts were back to normal and a pneumomediastinum was not detectable on chest X-ray. On day 7, the patient still required oxygen support, but was otherwise vitally stable. Echocardiography showed minimal interval increases of the pericardial effusion and no signs of cardiac tamponade. On day 9, she continued to improve clinically, and her vitals were within normal range. She required no oxygen support, and the repeated echocardiography showed interval improvement of the pericardial effusion. Gastrostomy feeding was resumed with minimal volume and incremented gradually. On day 13, an upper gastrointestinal contrast study showed no signs of contrast leakage or extravasation, suggesting that the perforation had resolved. The patient tolerated full gastrostomy feeding requirement, and total parenteral nutrition was discontinued. On day 15, an echocardiogram showed complete resolution of pericardial effusion. The patient finished her antibiotics course and was discharged home in stable condition with full oral feeding. She required five sessions of interval esophageal dilation, which were performed with rigid esophagoscopy by a pediatric surgeon and went uneventfully. Currently, the patient is 5 years old and is followed-up annually by our clinic. She is in complete remission, tolerating regular oral diet without signs or symptoms of esophageal stricture.

## Discussion

Acquired esophageal strictures are known complications in patients with cancer. Multiple factors can contribute to the outcome of an esophageal stricture in cancer patients, including their immune-compromised status, which makes them vulnerable to opportunistic infections, or chemotherapeutic agents and radiotherapy side effects, which can lead to mucositis. 3 Clinical presentation includes symptoms of dysphagia for liquid and solid food, regurgitation, cough, and poor oral intake. 4 The diagnosis of esophageal stricture can be confirmed radiologically by upper gastrointestinal series and/or diagnostic endoscopy. The management strategy must take into consideration the general condition of the patient and the severity, quantity, and site of strictures.

Dilatation of the esophagus remains the first line of treatment. 5 The balance of benefits and risks of intervention should be discussed within the multidisciplinary team managing the patient and with the family. Failure to dilate the stricture is not common and is considered an indication to proceed with surgical correction of the esophagus. 2 Surgical intervention to correct the stricture carries a risk of developing recurrent strictures, and patients may require further dilatation. 6

Esophageal leak postsurgical anastomosis is a predisposing factor in developing a stricture, 7 the treatment of which depends on the size of the leak but is most often conservative. 8 Our patient developed a postoperative leak, which was treated conservatively but developed a moderate esophageal stricture, which was managed later by dilatation. Esophageal dilatation is a common procedure among pediatric surgeons. Because it carries a low risk of postoperative complications if the procedure is uneventful, the procedure is usually performed in day surgery units, and the patient is discharged home after postoperative observation. 9

The dilatation technique depends on performer preference. The most commonly used technique is rigid dilatation, followed by a combination of rigid and balloon dilatations and balloon dilatation alone. 10 The reported success rate is up to 97.2%. 11 The frequency and time interval between esophageal dilatation depend on the patient's pathogenic cause and response to treatment and the surgeon's expertise and preference. Another study reported stricture resolution after a mean number of 3.2 dilatations (range, 1–7) with a success rate of 87%. 7

Esophageal perforation is a rare complication during dilatation. In a study in which 1,128 dilatations were performed, only 11 (0.98%) perforations were reported. 10 Another study reported a similar rate (0.9%) in 648 dilatation sessions. 11 Therapeutic instrumentation of the esophagus carries a higher risk of perforation compared with diagnostic instrumentation. 12

There are multiple options to treat esophageal perforation if detected during the procedure, and most of the reported cases were treated conservatively. 7 10 12 The management of esophageal perforation in pediatric patients is mainly conservative, which leads to spontaneous healing of the perforation, including drainage of intrathoracic collection, restrictive enteral intake, and ensured coverage with wide-spectrum antibiotics along with hydration and nutrition support. 13

To our knowledge, esophageal perforation into the pericardial space has not been reported in such a scenario although one case was found to have pneumopericardium evident in routine postdilation X-ray and was treated conservatively without details about the patient's clinical scenario or management approach. 13 Another case was reported to have signs of low cardiac output syndrome after ingestion of a foreign body and was found to have cardiac tamponade secondary to a pericardial–esophageal fistula, which was treated with surgical evacuation, drainage, and ligation of the fistula in the pericardium intraoperatively. 14

We think that the patient's history of severe mucositis, esophagitis, and acute pericarditis acted as a disposing factor by disturbing the normal anatomy of the mediastinum and led to esophageal perforation, which affected the pericardium and caused pericardial effusion. The treatment options in such scenarios must be discussed among the intensive care team, cardiac surgeon, and pediatric surgeon to decide the most appropriate intervention. Management might include observation, interventional pericardiocentesis, or surgical pericardiocentesis.

We believe that for patients who have a higher risk of abnormal mediastinal anatomy or morphologic irregularities due to previous severe inflammatory processes, such as in our patient, the decision to discharge the patient home on the same day of the procedure can be deliberated.

Once esophageal perforation is suspected, the initial management of wide-spectrum antibiotics and maintenance of good hydration status can prevent fatal consequences. If a perforation to the pericardium is detected, treatment can be conservative, with close observation for any signs of pericarditis or cardiac tamponade and immediate intervention if the patient's condition deteriorates and requires drainage.

## Notes

New Insights and Importance for a Pediatric Surgeon This case report illustrates the possibility of life-threatening complication during esophageal dilatation, the importance of high clinical suspicion for diagnosis and share our experience of the treatment by close observation and conservative management.
